# Elution of Monomers from an Infiltrant Compared with Different Resin-Based Dental Materials

**DOI:** 10.3290/j.ohpd.a43354

**Published:** 2020-07-04

**Authors:** Hendrik Meyer-Lueckel, Christian Hartwig, Hans G Börner, Julian Lausch

**Affiliations:** a Professor, Department of Restorative, Preventive and Pediatric Dentistry, University of Bern, Bern, Switzerland. Designed and planned the study; wrote and revised the manuscript.; b Postgraduate Student, Department of Operative and Preventive Dentistry, Charité – Universitätsmedizin Berlin, Berlin, Germany. Designed and planned the study; prepared the samples, performed the measurements and statistical analysis; revised the manuscript.; c Professor, Laboratory for Organic Synthesis of Functional Systems, Department of Chemistry, Humboldt-Universität zu Berlin, Berlin, Germany. Designed and planned the study; revised the manuscript.; d Postgraduate Student, Department of Restorative, Preventive and Pediatric Dentistry, University of Bern, Bern, Switzerland. Prepared the samples, performed the measurements and statistical analysis; wrote and revised the manuscript.

**Keywords:** caries, composites, elution, infiltration, TEGDMA

## Abstract

**Purpose:**

Low-molecular weight residuals eluting from dental materials may contribute to local and systemic adverse effects. Therefore, the aim of the present study was to compare triethylene glycol dimethacrylate (TEGDMA)-based commercial infiltrant with different conventional resin-based materials regarding their release of monomers.

**Materials and Methods:**

Cylindrical blocks (n = 10) of either two sealants (Helioseal, Delton FS+), a composite (EcuSphere), an adhesive (Teco) and an infiltrant (Icon) were prepared. Additionally, 20 artificial lesions (depths ≥100 µm) were created in bovine enamel and after etching with phosphoric acid infiltrated with the infiltrant. Except for 10 infiltrated lesions, all other specimens were polished. Each specimen was stored in 1 ml distilled water (elution medium) for 240 h. The medium was renewed in logarithmical divided time periods (4.5 min–76 h).

**Results:**

Total concentrations of eluted monomers within 240 h from the cylindrical specimens were 0.04–0.09 mg/ml (p >0.05; Mann–Whitney test). Unpolished infiltrated specimens showed significantly higher monomer concentrations compared to all other groups, whereas polishing of specimens resulted in significantly lower concentrations (p <0.05; Mann–Whitney test).

**Conclusion:**

It can be concluded that release of monomers was low in general, but for infiltrated lesions it was considerably reduced by surface polishing reaching similar values as for commonly used monomer-containing dental materials. Thus, adverse effects by the use of an infiltrant are not expected, but polishing of the infiltrated area should be considered.

Frequently used resin-based dental materials consist of a mixture of high- and low-molecular monomers that are hardened by light-curing through free radical polymerisation processes.^2,3^ While Bisphenol-A-glycidyl-methacrylate (BisGMA) and 1.3 glycerol-dimethacrylat (G-1.3-DMA) are high viscosity monomers, triethylene glycol dimethacrylate (TEGDMA) and hydroxyethylmethacrylate (HEMA) are low-viscosity monomers for dental applications. Despite the ubiquitous popularity of these monomers, increasing concerns are discussed that leaching of residual monomers from dental resins might cause local or systemic allergic or even toxic effects.^9,15,25^ Systematic intake might occur due to ingestion through the gastrointestinal tract, via inhalation through the lungs or by diffusion through the dentine tubules into the pulp.^10,16^ Furthermore, cellular metabolisms up to vital cell mechanisms might be affected by those low-molecular weight entities that come into contact with local tissues over time.^15^

Depending on the components and composition of dental resin precursor mixtures as well as the application and hardening procedures applied, the degree of monomer to polymer conversion can vary between 65% and 75%.^9^ Quantitative conversion is practically not straightforward to be achieved due to diffusion limitations that occur in the hardening procedure. Thus, short-term release of residual, non-polymerised monomers occurs by diffusion during the photo curing step, and monomer leaching takes place over time after the hardening of the dental specimen is finished.^1,5,12,25^

Thus, measurable amounts of monomers elute from the surfaces of the dental material into aqueous environments such as the oral cavity.^11^ The release of monomers from conventional dental resins composed of composite fillers, sealants and adhesives is an established problem and possible adverse effects on cells, tissues or even systemic mechanisms have been previously described.^2,8,11,25^ However, in contrast to these frequently used dental materials, less is known about the release of residual monomers from a novel class of low-viscosity resins referred to as infiltrants. The technique of infiltration aims at penetrating low-viscosity resin into the pores of early enamel lesions in order to arrest or delay advancement of caries processes.^17^ After a pretreatment with 15% hydrochloric acid gel for 2 min to erode the surface layer that normally covers a lesion, infiltrants penetrate several hundred micrometers into the lesion body and hardening within the lesion takes place by light-curing. Besides the pretreatment, a successful caries infiltration strongly demands low viscosity to fulfill the penetration properties of the infiltrant.^23^ Therefore, infiltrants almost exclusively consist of low-molecular monomers with TEGDMA contents of up to 95% and do not include microfillers.^22^ However, not much is known about leaching of residual monomers from infiltrant penetrated enamel lesions.

Thus, the aim of the present study was to evaluate the leaching of residual monomers from pure blocks of the low-viscosity infiltrant compared to the different conventionally used resin-based dental materials. In addition, the elution of monomers from infiltrant-penetrated artificial lesions was analysed. It was hypothesised that the release of monomers from an infiltrant would be significantly higher compared to other dental resins.

## Materials and Methods

All resin-based dental materials used in the present study are shown in Table 1. Two different kinds of specimen (a, pure blocks of resin, b, infiltrated enamel caries lesions) were evaluated in the present study. From each resin and enamel caries specimen cylindrical samples were made with the help of metallic moulds (depth 1 mm, diameter: 3 mm) and left in these for the following experiments, so only the upper surface was exposed to the elution medium. All resins were light cured for 60 s (Ortholux Luminous, 3M Oral Care, Neuss, Germany; irradiance: 1600 mW/cm^2^) and polished (Mikroschleifsystem 400 cs with abrasive paper 600, 800, 1200, Exakt Apparatebau, Norderstedt, Germany).

**Table 1:** Resin-based dental materials used in the present study

Material	Manufacturer	Type	Composition*
Helioseal	Ivoclar Vivadent, Schaan, Lichtenstein	Fissure sealant	BisGMA, TEGDMA, titanium oxide, catalyst, stabiliser
Delton FS+	Dentsply, Konstanz, Germany	Fissure sealant	BisGMA, TEGDMA, barium-aluminium-silikat-glass, titanium oxide, natrium fluoride, catalyst, stabiliser
Teco	DMG, Hamburg, Germany	Adhesive	G-1.3-DMA-based matrix, HEMA, initiators, additives, pigment
EcuSphere	DMG, Hamburg, Germany	Composite	G-1.3-DMA-based matrix, barium glass, catalyst, additives, pigment
Icon	DMG, Hamburg, Germany	Infiltrant	TEGDMA-based resin matrix, initiators, additives

*Manufacturer’s data sheets; BisGMA, bisphenol-A-glycidyl-methacrylate; G-1.3-DMA, 1.3 glycerol-dimethacrylat; TEGDMA, triethylene glycol dimethacrylate; HEMA, hydroxyethylmethacrylate.

From extracted bovine incisors 20 enamel slices (diameter: 3 mm) were prepared (Band Saw 300cl, Exakt Apparatebau, Norderstedt, Germany) and mounted within metallic moulds using instant glue (Dentalsekundenkleber, Multident, Berlin, Germany). In order to create artificial lesions, enamel specimens were stored in 5 L of a demineralising solution containing 5 mM acetic acid, 3 mM CaCl_2_·H_2_O, 3 mM KH_2_PO_4_ and 4 µM methylhydroxydiphosphonate (Merck, Darmstadt, Germany) (pH 4.95, 37°C) for 21 days.^4^ The pH value was controlled daily and corrected with hydrochloric acid solution (10%) (Merck, Darmstadt, Germany) or potash lye (19%) (Merck, Darmstadt, Germany). After demineralisation the artificial lesions were etched with 37% phosphoric acid gel (Email Preparator, Ivoclar Vivadent, Schaan, Liechtenstein) for 30 s, rinsed (30 s) and dried (compressed air 30 s). After this procedure, the infiltrant (DMG, Hamburg, Germany) was applied (120 s) and light cured (60 s) (Ortholux Luminous, 3M Oral Care, Neuss, Germany). Only half of the infiltrated lesions were polished (n = 10) using abrasive paper (Mikroschleifsystem 400 cs, with abrasive paper 600, 800, 1200, Exact Apparatebau, Norderstedt, Germany). All other infiltrated samples (n = 10) remained unpolished.

Each of the blocks of pure resin as well as the infiltrated enamel lesions were stored in 1 ml distilled water (elution medium) for 240 h. For the pure blocks of resin, the elution medium was renewed in logarithmical divided time periods (4.5 min, 14.5 min, 2.5 h, 7.5 h, 24 h and 76 h). For the infiltrated enamel lesions, the elution medium was not renewed but the concentration of released monomers was measured after 240 h. The elutes of residual monomers (TEGDMA, BisGMA, G-1.3-DMA and HEMA) were determined by high-performance liquid chromatography (HPLC) on a RP-18 phase detecting at 205 nm and 220 nm (LCMS QP8000alpha, Shimadzu, Kyoto, Japan). The parameters that showed a clear separation of peaks for each of the tested monomers were defined before the main analysis (flow speed: 0.4 ml/min, cycle time: 45 min). In order to quantify each monomer a calibration standard curve of peak areas and monomer concentrations in acetonitrile/water solution (80%/20%) (Merck, Darmstadt, Germany) was created at known concentrations (TEGDMA: 0.01–0.25 mg/ml, HEMA/BisGMA/G-1.3-DMA: 0.01–0.4 mg/ml). The HPLC-measurement was performed using LCMS Solution PostRun software (Shimadzu, Kyoto, Japan). The concentration at the given time periods as well as a cumulative concentration (after 240 h including the six renewals of elution medium) were measured for each type of monomer.

Data were checked for normal distribution (Shapiro-Wilk test). Differences between the groups were analysed using Kruskal-Wallis and Mann-Whitney test. The level of statistical significance was set to 5% for all tests (SPSS Version 20.0, SPSS, Munich, Germany).

## Results

Only one sample was destroyed during the preparation for the HPLC-measurement. Unbound low-molecular TEGDMA was released from the infiltrant and the two fissure sealants but not from the adhesive or the composite. HEMA was leached from the adhesive only. For all of these materials highest amounts of TEGDMA and HEMA were detected within the early 4.5 min time interval (Table 2).

**Table 2:** Release of monomers from the various dental resins

Monomer	Dental material	Detection time	Percentage release after 4.5 min	Percentage release after 24 h	Entire release after 240 h (mg/ml)
BisGMA	Helioseal	2.5 h	0%	47%	0.0047 mg/ml
	Delton FS+	2.5 h	0%	70%	0.0060 mg/ml
G-1.3-DMA	Ecusphere	7.5 h	0%	47%	0.06 mg/ml
	Teco	7.5 h	0%	28%	0.07 mg/ml
TEGDMA	Infiltrant	0–4.5 min	66%	89%	0.05 mg/ml
	Helioseal	0–4.5 min	31%	99%	0.06 mg/ml
	Delton FS+	0–4.5 min	21%	62%	0.06 mg/ml
HEMA	Teco	0–4.5 min	18%	88%	0.02 mg/ml

BisGMA, bisphenol-A-glycidyl-methacrylate; G-1.3-DMA, 1.3 glycerol-dimethacrylat; TEGDMA, triethylene glycol dimethacrylate; HEMA, hydroxyethylmethacrylate.

High molecular BisGMA was released from the two fissure sealants, the G-1.3-DMA was detected for the composite and the adhesive but both were not leached from the infiltrant. The BisGMA and G-1.3-DMA were not seen within the early time intervals. BisGMA was detected after 2.5 h the G-1.3-DMA after 7.5 h (Table 2).

**Fig 1:**
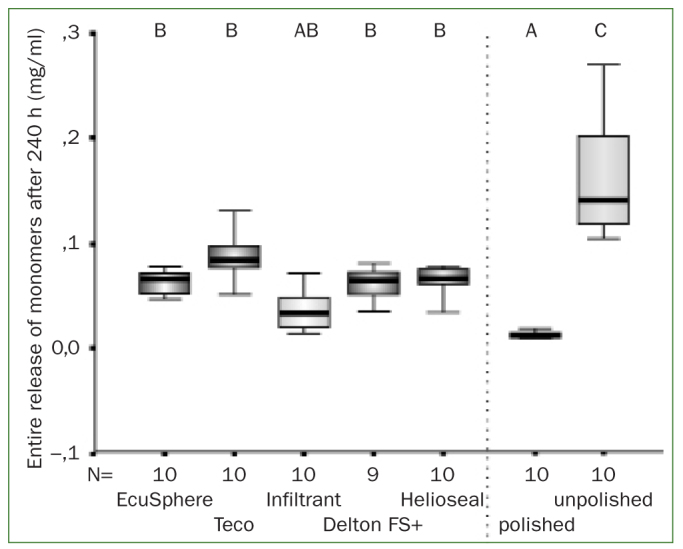
Cumulative release of monomers (mg/ml) after 240 h (box-and-whisker plots with quartiles and medians) for the pure resins as well as for polished and unpolished infiltrated caries lesions. Statistically significant differences between various materials are indicated by different letters (p <0.05; Mann–Whitney test); n = number of samples.

The overall amounts of leachable monomers after 240 h did not differ significantly between the various resin materials (p > 0.05). However, very high amounts of monomers were leached from resin-infiltrated enamel lesion. Interestingly, polishing had a strong effect on the released residual monomers as infiltrated but unpolished lesions released significantly higher amounts of monomers compared to polished samples (Fig 1; p <0.05).

## Discussion

In the present study all dental materials released some of their monomers. The study hypothesis that the infiltrant would elute significantly higher concentrations of monomer compared with other dental materials could not be confirmed. However, the study showed that unpolished infiltrated enamel lesions released significantly higher concentrations of TEGDMA compared with polished ones.

Previous studies utilised deionised water or buffer as elution medium but human or artificial saliva, which would correspond more closely to the realistic model system for elution did not show reliable HPLC-measurement results. Despite the fact that HPLC is applicable to determine small amounts of substances^21^ the presence of a complex mixture in the saliva media prevents reproducible measurements. Therefore, distilled water was chosen as elution medium. Since the study aimed at showing relative differences between the infiltrant under various conditions compared with commonly used dental resin-based materials this methodological shortcoming seems to be a minor issue.

In the present study artificial and rather shallow lesions (lesion depth ≥100 µm) were used. Compared with natural lesions a partial breakdown of the surface of artificial lesions was described previously, if a pretreatment with 15% hydrochloric acid gel for 2 min (recommended for the clinical use of caries infiltration technique) was used.^20^ To improve the access for the infiltrant into artificial lesions a pretreatment with 37% phosphoric acid seems to be sufficient to remove the surface layer that normally covers the subsurface lesion body.^13,18^ Thus, the shorter etching with 37% phosphoric acid for 30 s was performed in the present study as well.

The elution of monomers in vitro strongly depends on the type and volume of the solvent. In contrast, to distilled water organic solvents (eg, ethanol, ethanol/water solutions) penetrate into the polymer matrix of dental resins and enlarge spaces between polymer chains resulting in a higher release of substrates.^19^ Further, the higher the volume of the surrounding solvent (eg, 1 ml per specimen) the lower the concentrations of released monomers at same extent of the specimen. Thus, depending on experimental conditions in vitro findings of monomer release about the same dental resin could differ between various elution studies.^6^ However, the results of the present study corroborate findings from comparable evaluations.^7,14^ that leaching of BisGMA or G-1.3-DMA probably do not reach concentrations that induce adverse effects in a clinical situation.

TEGDMA and HEMA (very flexible, low molecular, low viscous) are frequently used in adhesives as well as in the infiltrant. The highest concentration of TEGDMA was detected within the first minutes after light-curing of the infiltrant and only very low concentrations were observed in later time intervals. Thus, in the short-term the main exposure from leached TEGDMA seems to occur during the treatment procedure itself. Even in the first minutes after application and light-curing the concentration of leached TEGDMA was in the same magnitude as those observed for other monomers. Previous studies showed highest releases of monomers from resins if an oxygen-inhibition layer was present on the outer surfaces of a material.^26^ In the present study an oxygen-inhibition layer was observed after light-curing of the infiltrant, as well. By polishing elution of monomers from the infiltrated enamel lesions was significantly reduced. Thus, for preventions of possible local adverse effects polishing of infiltrated enamel lesion could be useful.

It was shown before that very low concentrations of TEGDMA might cause cell death due to necrosis after a time of exposure of 24 h.^24^ If TEGDMA reaches the pulp, lower concentrations could also inhibit phosphatidyllinositol-3-kinase that might result in a high amount of apoptotic cells.^25^ In the present study only unpolished infiltrated enamel lesions released relevant concentrations of TEGDMA. Since the infiltrant is used for enamel but not for dentine lesions TEGDMA should not diffuse into the pulp. Furthermore, contact times of 24 h seem to be improbable since TEGDMA was released from an infiltrant almost completely within the first minutes after treatment. However, local or systemic effects of leached monomers were not evaluated in this study. Nonetheless, as seen for sealants, composites or adhesives also the infiltrant released only very low concentrations of monomers.

The present investigation indicate that only low concentrations of released monomers could be found for the frequently used dental materials and the infiltrant. Polishing of infiltrated enamel lesions seems to be suitable to obtain low monomer concentrations, similar to those of cylindrical blocks of the infiltrant and other common resin materials. Thus, it seems to be improbable that the infiltrant cause adverse effects more seriously compared with other resin-based dental materials in clinical practice.
